# Synthesis and structure of undoped and indium-doped thermoelectric lead telluride nanoparticles

**DOI:** 10.1186/1556-276X-9-227

**Published:** 2014-05-08

**Authors:** Kamal Kadel, Latha Kumari, Xuewen Wang, Wenzhi Li, Jian Yu Huang, Paula Polyak Provencio

**Affiliations:** 1 Department of Physics, Florida International University, Miami, FL 33199, USA; 2 Sandia National Laboratories, Albuquerque, NM 87185, USA

**Keywords:** Lead telluride, Nanostructure, Solvothermal/hydrothermal synthesis, First principle calculation

## Abstract

Undoped and indium (In)-doped lead telluride (PbTe) nanostructures were synthesized via solvothermal/hydrothermal route. The crystalline structure of the as-prepared undoped and In-doped PbTe samples was examined by X-ray diffraction (XRD) which indicated the formation of face-centered single-phase cubic crystal. A first principle calculation on indium doping shows that the indium atoms are more likely to replace lead (Pb) rather than to take the interstitial sites. Laser-induced breakdown spectroscopy (LIBS) analysis confirms that indium is incorporated into the PbTe matrix of the indium-doped PbTe samples. The effects of surfactant and synthesis temperature on the structure and morphology of the undoped PbTe were also investigated; it was found that PbTe nanostructures synthesized with the addition of surfactants exhibited uniform shapes and their size increased with the synthesis temperature.

## Background

Though solid-state thermoelectric (TE) materials are considered as potential candidates for their application in power generating and refrigerating devices [1], the low efficiency of the TE materials limits their practical application [2]. Nanostructured materials are drawing more attention due to their potential applications in thermoelectrics with high efficiency. Theoretical predictions and experimental results indicate that low-dimensional TE materials can exhibit high thermoelectric efficiency [3-5]. The efficiency of TE materials can be defined by dimensionless thermoelectric figure of merit (ZT), ZT = (*S*^*2*^*σ*/*κ*)*T*, where *S* is the Seebeck coefficient, *σ* is the electrical conductivity, *κ* is the thermal conductivity, and *T* is the absolute temperature at which the figure of merit is measured. The quantity *S*^2^*σ* is most commonly referred as power factor. Increase in power factor and decrease in thermal conductivity are required to enhance the ZT value. Nanostructures can induce the reduction of thermal conductivity due to the enhanced phonon scattering by the interface or the boundary and the increment in power factor via quantum confinement of electrons [4]. According to Slack [6], semiconductors having narrow band gap and high mobility carriers are best suited for thermoelectric materials. Lead telluride (PbTe) is a narrow band gap semiconducting material and has great applications in thermoelectric devices, IR photoelectrics [7], and IR laser devices [8]. PbTe is considered as one of the best thermoelectric materials which can be efficiently employed as a power generator in the medium and high temperature range (450 to 800 K) [9]. It is shown theoretically and experimentally that the TE property of PbTe can be improved by doping it with some donor or acceptor atoms. Recently, there has been renewed research interest in PbTe after Heremans et al. [7] reported the enhancement of the Seebeck coefficient of PbTe through the distortion of electronic density of states by doping it with thallium. The electric property of PbTe can vary significantly when it is doped with group IIIA elements, such as In and Ga, which generate a deep lying impurity level in IV-VI compounds [10]. A previous work by Dashevsky et al. [11] reported a higher ZT value of about 0.92 at 700 K for a functionally graded indium-doped single crystal of PbTe.

PbTe nanostructures have been synthesized using various techniques. Beyer et al. [12] reported an enhanced thermoelectric efficiency of molecular beam epitaxially (MBE) grown superlattices based on PbTe. Palchik et al. [13] synthesized PbTe from solutions under microwave radiations. Earlier works also reported the synthesis of 3-D structures of PbTe such as dendrite-like structures via electrochemical deposition [14] and sponge-like structures from sonochemistry [15]. Among the various synthesis techniques employed for the formation of PbTe nanostructures, the solvothermal/hydrothermal process has attracted much interest due to the advantage of high yield, low synthesis temperature, high purity, and high crystallinity. Zhu et al. reported the synthesis of PbTe powders using alkaline reducing solvothermal route [16] and the synthesis of PbTe three-dimensional hierarchical superstructures via an alkaline hydrothermal method [17]. The solvothermal/hydrothermal technique produces various PbTe nanostructures such as nanotubes [18,19], nanospheres [20], and nanoboxes [21]. In this work, we report the synthesis of undoped and In-doped PbTe nanostructures using the solvothermal and hydrothermal routes in alkaline solution medium with or without a surfactant at different temperatures and reaction time durations. We have explored the synthesis of the undoped and In-doped PbTe nanostructures using a water/glycerol mixture as a solvent, which, to the best of our knowledge, has not been previously reported. The morphology and crystal structure of the as-synthesized undoped and In-doped PbTe nanostructures have been discussed in detail. Laser-induced breakdown spectroscopy (LIBS) analyses were conducted to investigate the indium incorporation into the PbTe matrix. A pseudo-potential first principle calculation was conducted to study the mechanism of indium doping into the PbTe matrix. In-doped PbTe is expected to enhance the thermoelectric property due to the increase in Seebeck coefficient through the distortion electron density of states near the Fermi level.

## Methods

Analytically pure lead nitrate (PbNO_3_), indium chloride (InCl_3_), and tellurium (Te) powder were used as precursor materials for the synthesis of PbTe and In-doped PbTe. These materials were put in the Teflon liner in the appropriate molar ratios according to the formula In_*x*_Pb_1-*x*_Te, where *x* = 0, 0.005, 0.01, 0.015, and 0.02. Then, 6.25 mmol of sodium hydroxide (NaOH) as a pH controlling agent, 2.6 mmol of sodium borohydrate (NaBH_4_) as a reducing agent, and 1 mmol of ethylenediaminetetraacetic acid (EDTA) as a shape-directing additive were added. Water was used as a solvent in the hydrothermal process; either ethanol or a mixture of glycerol and water in 1:3 volume ratio was used as solvent for the solvothermal route. Later, the Teflon liner was filled up to 80% of its total volume with the solvent and was placed in an ultrasonicator for 30 min to obtain a uniform reaction mixture. After sonication, the Teflon liner was placed in an autoclave and sealed tightly. Then, the autoclave was heated in the furnace at 140°C and 200°C for 24 h. After synthesis, the autoclave was allowed to cool down to room temperature naturally. A black precipitate was collected, and then vacuum filtered, rinsed with ethanol and distilled water several times repeatedly, and dried at 120°C in vacuum for 4 h. The above synthesis process was repeated with the addition of 1 mmol each of cetyltrimethylammonium bromide (CTAB), sodium dodecyl sulfate (SDS), and Triton X-100 as cationic, anionic, and non-ionic surfactants/capping agents, respectively, at 140°C for 24 h in water/glycerol solution (3:1 volume ratio). The PbTe nanostructures synthesized without surfactants at 140°C and 200°C for 24 h in ethanol are termed as PbTe-1 and PbTe-3 and in the water/glycerol mixture are named as PbTe-2 and PbTe-4, respectively. In_*x*_Pb_1-*x*_Te (*x* = 0.005, 0.01, 0.015, and 0.02) synthesized at 140°C for 24 h in water/glycerol solution are named as In005PbTe, In01PbTe, In015PbTe, and In02PbTe, respectively.

X-ray diffraction (XRD) measurements were carried out using a Siemens D5000 diffractometer equipped with a Cu anode operated at 40 kV and 40 mA (Siemens AG, Karlsruhe, Germany). The XRD patterns were collected with a step size of 0.01° and a scan rate of 1 step per second. Surface morphology analysis was performed by a field emission scanning electron microscope (SEM, JEOL JSM-6330 F, 15 kV; JEOL Ltd., Tokyo, Japan). Transmission electron microscopy (TEM), selected-area electron diffraction (SAED) patterns, and energy dispersive X-ray spectroscopy (EDS) spectrum were obtained from a FEI Tecnai F30 apparatus (FEI Co., Hillsboro, OR, USA) operated at an accelerating voltage of 300 kV with a point-to-point resolution of 2 Å. LIBS analyses were conducted on a RT100HP system (Applied Spectra, Fremont, CA, USA), equipped with a 1,064-nm ns-Nd:YAG laser. The detector has a CCD linear array (Avantes, Broomfield, CO, USA) with possible gate delay adjustment from 50 ns to 1 ms with 25-ns step resolution and a fixed integration time of 1.1 ms. Data interpretation and data analysis were conducted with TruLIBS™ emission database and Aurora data analysis software (Axiom 2.1, Applied Spectra, CA, USA).

A first principle calculation was conducted to investigate the indium doping into the PbTe matrix. We first calculated the lattice constant of PbTe in its NaCl structure. Then, we constructed a simple cubic (SC) 2 × 2 × 2 supercell with 32 PbTe units and used the same lattice constant for further calculation of substitution energy and interstitial insertion energy.

## Results and discussion

**Figure 1:**
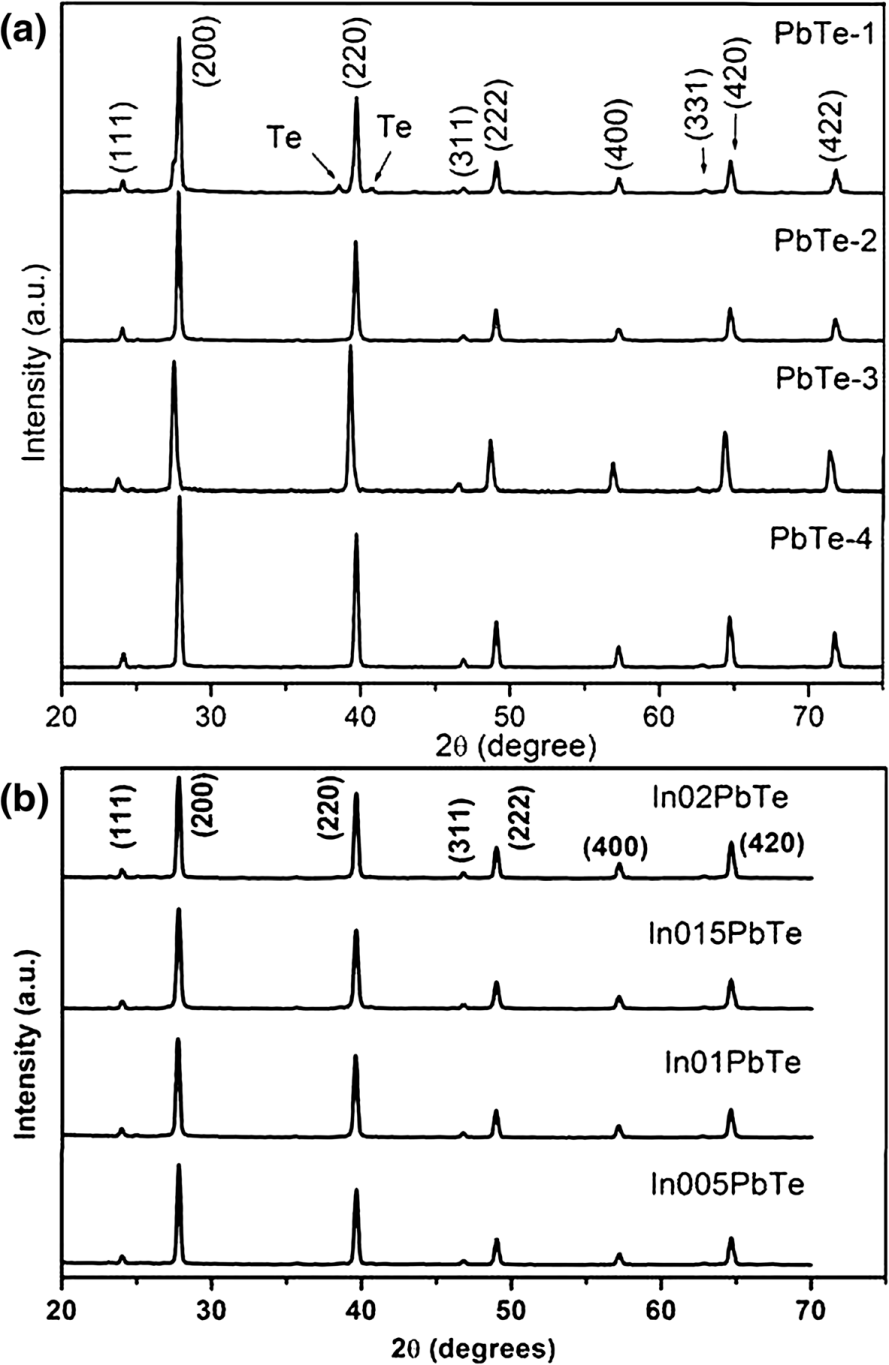
**XRD patterns of undoped and In-doped PbTe samples. (a)** XRD patterns of the as-prepared undoped PbTe samples synthesized without surfactants for 24 h: PbTe-1 at 140°C in ethanol solution, PbTe-2 at 140°C in water/glycerol solution, PbTe-3 at 200°C in ethanol, and PbTe-4 at 200°C in water/glycerol solution. **(b)** XRD pattern of In-doped PbTe samples synthesized at 140°C for 24 h: In005PbTe, In01PbTe, In015PbTe, and In02PbTe synthesized in water/glycerol solution.

Figure 1 shows the XRD patterns of the as-prepared samples. Figure 1a shows the XRD pattern of undoped PbTe samples PbTe-1, PbTe-2, PbTe-3, and PbTe-4. All the diffraction peaks in the XRD patterns can be indexed as a face-centered cubic PbTe (JCPDS: 78-1905) [16] which confirms the crystalline phase purity of the as-synthesized PbTe. The strong (200) diffraction peak represents the prominent growth orientation of PbTe nanostructures along the [200] direction. The sharp peaks in the XRD profiles indicate the high crystallinity of the PbTe sample. However, the XRD profile for PbTe-1 sample shows two weak peaks on either side of the (220) peak, which can be attributed to the presence of some elemental Te [22]. The residual Te indicates that the synthesis in ethanol at relatively low temperature (140°C) is an incomplete reaction. The results indicate that if ethanol is used as the solvent, a high reaction temperature is needed to promote a complete reaction and achieve high-purity PbTe (see the XRD pattern labeled PbTe-3 in Figure 1a). Furthermore, if a water/glycerol mixture is utilized as the solvent, pure phase of PbTe can be formed at either a low temperature of 140°C (see the XRD pattern labeled PbTe-2 in Figure 1a) or a high temperature of 200°C (see the XRD pattern labeled PbTe-4 in Figure 1a). It is clear that solvent of a water/glycerol mixture facilitates the reaction. Because only water/glycerol mixture yields a pure phase of PbTe at all synthesis conditions including lower temperature (140°C) synthesis, our all indium-doped samples were prepared in water/glycerol solution at 140°C for 24 h, which are the same conditions used for synthesizing undoped sample PbTe-2.

**Figure 2:**
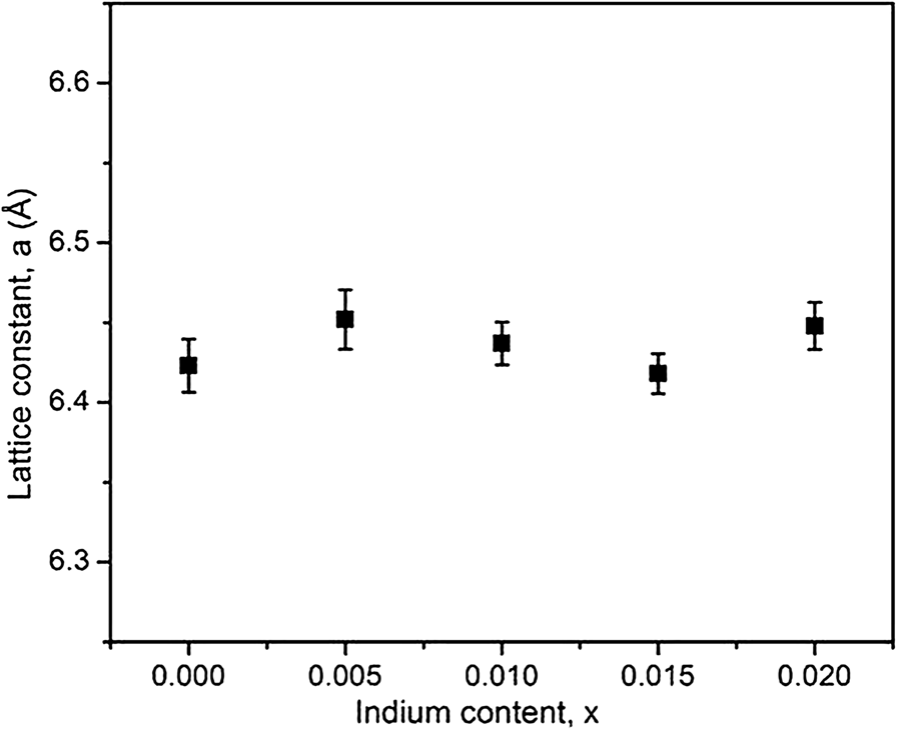
**Graph of lattice constant versus doping level of indium in In-doped PbTe samples.** The samples were synthesized at 140°C for 24 h in water/glycerol solution.

Figure 1b represents the XRD patterns of In-doped PbTe (In005PbTe, In01PbTe, In015PbTe, and In02PbTe) synthesized at 140°C for 24 h in water/glycerol solution. All the diffraction peaks belong to the same face-centered cubic structure as that of PbTe and the very sharp peaks indicating the high crystallinity of the as-synthesized In-doped PbTe samples. XRD patterns do not show any peaks corresponding to elemental indium, indicating that indium is likely doped in PbTe. Lattice constants of undoped (PbTe-2) and indium-doped samples were calculated from the respective XRD profiles using Bragg's law and were tabulated in Table 1. As indium atoms are smaller in diameter than Pb atoms, lattice constants of the In-doped PbTe are expected to decrease. However, the lattice constants for undoped and all indium-doped PbTe samples are almost the same (average value approximately 6.434 Å) which is in agreement with the reported value for undoped cubic PbTe (6.454 Å, JCPDS: 78-1905). Figure 2 shows the variation of lattice constant of our indium-doped PbTe samples with different molar fractions of indium doping prepared at 140°C for 24 h in water/glycerol solution. The graph indicates that our PbTe samples do not exhibit any consistent decrease in lattice constant with the doping level of indium. Samoylov et al. [23] reported a very small decrease (on the order of 10^-3^ Å) in the lattice constant of In-doped PbTe films within the molar fraction interval of 0 < *x* < 0.064 of indium. This decrease is 1 order of magnitude smaller than the uncertainty in lattice constant in our samples (see Table 1). Another work by Belokon et al. [24] also reported almost constant lattice parameter with the doping level of indium up to 2 at% of indium doping. The bigger uncertainty in the lattice constant calculation in our samples can be attributed to the limit of the method used in the calculation. The possible minute change in lattice constant with the indium content is beyond the detectable limit of our XRD system.

**Table 1:** Lattice constants of undoped and In-doped PbTe samples

**Doping type**	**Sample name**	**Lattice constant, Å**
Undoped	PbTe-2	6.423 ± 0.017
Doped	In005PbTe	6.452 ± 0.019
	In01PbTe	6.437 ± 0.014
	In015PbTe	6.418 ± 0.013
	In02PbTe	6.441 ± 0.015

To further investigate the doping mechanism, we studied the favorability of indium atom to substitute Pb by conducting the pseudo-potential first principle calculations using a single cubic 2 × 2 × 2 supercell with 32 units of PbTe. We first started with 64-atom Pb_32_Te_32_ cell to calculate the lattice constant of PbTe crystal. The calculated value of the lattice constant is found to be 6.33 Å which is in close agreement with the reported value for cubic PbTe, 6.454 Å (JCPDS: 78-1905). This is followed by calculation of the formation energy for substitution with one indium in the 2 × 2 × 2 supercell (1.5 at% of In) which is slightly higher in indium level compared to our highest doped experimental sample In_0.02_Pb_0.98_Te (1.0 at%). The formation energy of the substitution is defined as *E*_sub_ = *E*(Pb_32_Te_32_) + *E*(In) - *E*(InPb_31_Te_32_) - E(Pb). The calculated value of the formation energy of the substitution is 3.21 eV which is larger than the calculated cohesive energy of indium crystal (*E*_in_), 2.52 eV. Since *E*_sub_ > *E*_in_, we can conclude that indium is highly favorable to substitute Pb into the PbTe for 1.5 at% doping level. This conclusion is consistent with the result we got from the XRD analysis of our In-doped PbTe samples. No indium phase is detected by XRD in our sample. We further calculated the formation energy of substitution for InPb_15_Te_16_ (3.12 at% of In) and InPb_7_Te_8_ (6.24 at% of In) in order to investigate the solubility of the indium into PbTe. It is found that formation energy for substitutions reduced to -0.6 and -1.17 eV, respectively, for 3.12 and 6.24 at% of indium doping. The reduced value of substitution energy indicates that substitution of Pb with indium becomes less favorable with the increased In doping concentration. The very large negative substitution energy, -1.17 eV for 6.24 at% of In doping, suggests that it is almost impossible for In to substitute Pb at such high doping level. This corresponds well with the solubility limit of In in PbTe. We have also tested In doping into interstitial sites of the PbTe lattice. At the most likely (0.25, 0.25, 0.25) interstitial site, the insertion energy comes to be 0.068 eV. From these energy calculations, as well as from our X-ray measurement, we can conclude that In doping, at our level of 1.5 at%, allows substitution on the Pb site.

Our conclusion is consistent with a previous first principle calculation of aluminum (Al) doping on PbSe [25], which also concluded that Al atoms prefer to replace Pb rather than to take interstitial sites. The reported band structure and density of states (DOS) calculation showed that upon low-level doping of Al, the enhanced density of states of PbSe near the Fermi energy is responsible for enhanced carrier density, which leads to higher conductivity. Since In doping to our PbTe sample allows substitution on the Pb site, we expect a similar effect on electronic properties of our PbTe samples upon doping.

**Figure 3:**
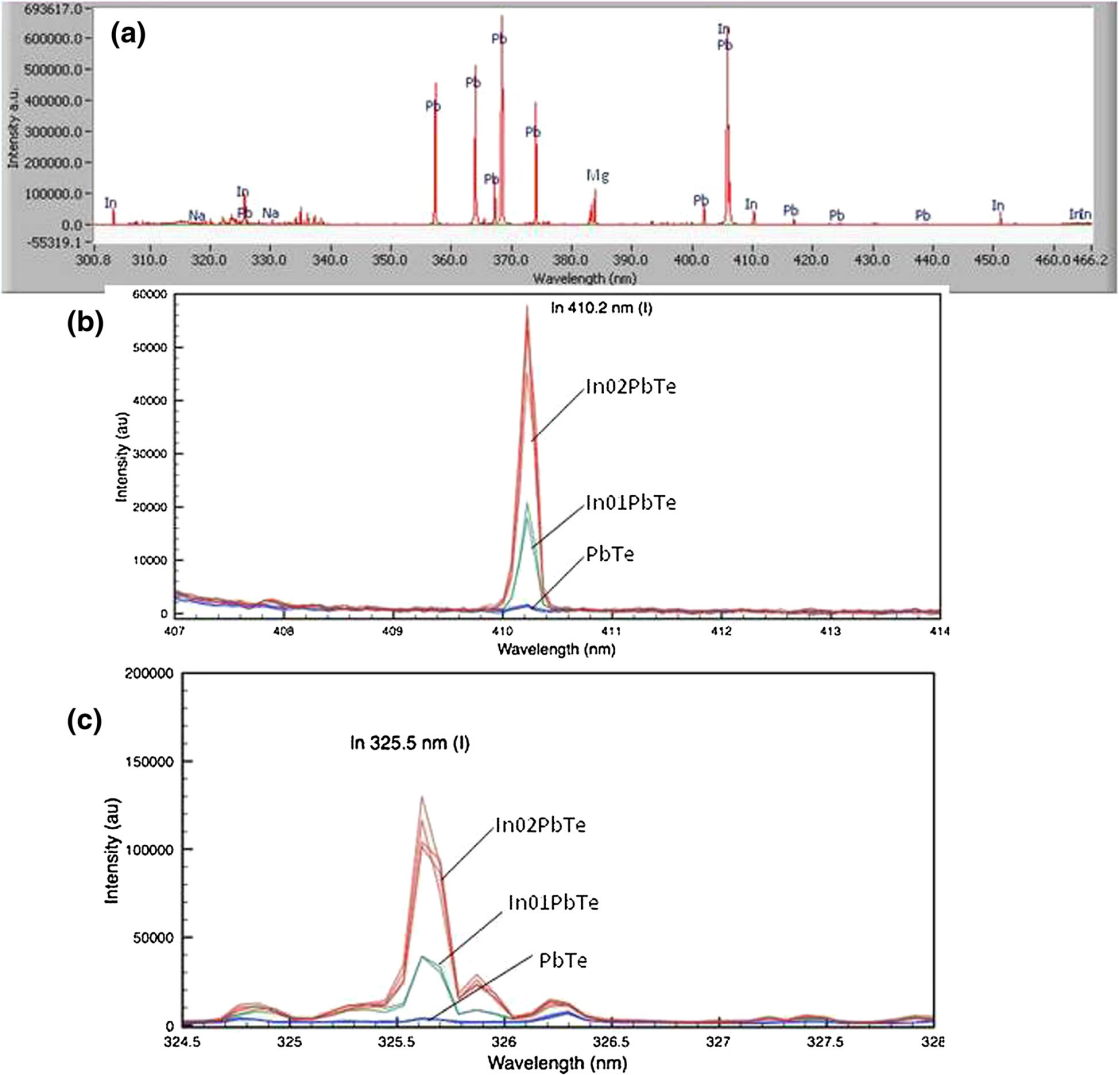
**LIBS spectra and emission lines. (a)** LIBS spectra of In02PbTe for selected range from 300 to 466 nm. **(b)** LIBS indium emission lines at 410 nm for samples PbTe-2 (blue), In01PbTe (green), and In02PbTe (red), respectively. **(c)** LIBS indium emission lines at 325 nm for samples PbTe-2 (blue), In01PbTe (green), and In02PbTe (red), respectively.

To further investigate the incorporation of indium into the PbTe matrix, the LIBS analyses were performed on the undoped (PbTe-2) and two indium-doped (In01PbTe and In02PbTe) samples, respectively. LIBS emission spectra were obtained in the wavelength range of 200 to 1,040 nm. The presence of indium in the samples In01PbTe and In02PbTe was confirmed by the detection of nine different emission lines at 256.0, 271.0, 275.4, 293.3, 303.9, 325.6, 410.2, 451.1, and 465.6 nm. Figure 3a shows typical spectra and some emission peaks detected for In and Pb on sample In02PbTe. Tellurium (Te) peaks were not detected due to the very high ionizing potential of Te which was beyond the operational range of the LIBS instrument. LIBS spectra also show some prominent impurity peaks of magnesium (Mg) which may have come from some trace amount of metal impurities (approximately 0.2%) present in the precursor materials (Te) used in the synthesis. Figure 3a is the LIBS emission spectra of In02PbTe for the selected range from 300 to 466 nm which shows the presence of atomic indium peaks at different wavelengths from 256.0 to 466 nm. Figure 3b,c shows the LIBS indium emission lines at 410 and 325 nm for undoped PbTe (blue), In01PbTe (green), and In02PbTe (red), respectively. Undoped PbTe does not show any indium peak at both the wavelengths, indicating the absence of indium. However, In01PbTe and In02PbTe samples show the presence of indium lines at 410 and 325 nm with almost linear increase in intensity with increasing indium content. The presence of multiple indium emission lines and linear increase in intensity from the samples In01PbTe and In02PbTe confirm the incorporation of indium into the PbTe matrix in doped samples. From the result of LIBS analyses, first principal energy calculations, and X-ray measurement, we can conclude that at the level of 1.5 at% doping, indium is doped in the PbTe matrix with the substitution on the Pb site.

**Figure 4:**
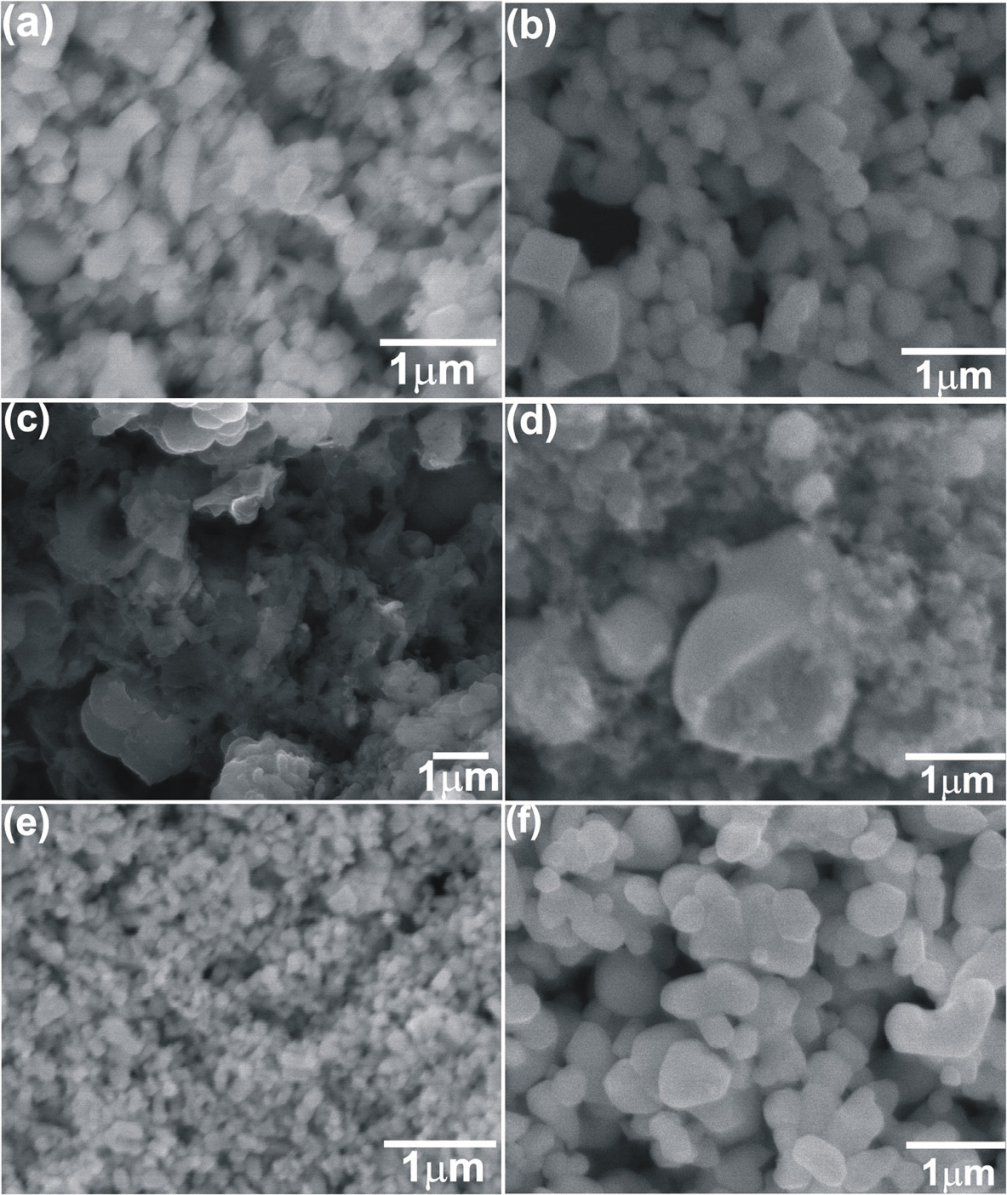
**SEM images of the PbTe samples prepared at 140°C and 200°C with different solvents.** SEM images of undoped PbTe nanoparticles prepared without surfactants for 24 h in ethanol at **(a)** 140°C and **(b)** 200°C, in water at **(c)** 140°C and **(d)** 200°C, and in water/glycerol solution at **(e)** 140°C and **(f)** 200°C.

Figure 4 shows the SEM images of the PbTe samples prepared at 140°C and 200°C with different solvents, respectively. Figure 4a is the SEM image of the sample prepared with ethanol as the solvent at 140°C for 24 h which shows particles with appreciably uniform shape and average particle size of about 200 nm. However, with ethanol at 200°C for 24 h (Figure 4b), particles grow larger to an average size of about 300 nm. For comparison, the synthesis of PbTe samples was attempted with water as the solvent. Figure 4c,d presents the SEM images of the PbTe samples synthesized with water as the solvent at 140°C and 200°C for 24 h, respectively. From the images, it is clear that the PbTe samples formed with water as the solvent have chunks with various shapes and sizes. The PbTe sample prepared at 200°C for 24 h with water as solvent (Figure 4d) shows nano- to micron-sized spherical particles along with irregularly shaped particles. This result indicates that water alone is not sufficient for the formation of uniform small-sized PbTe nanoparticles. Figure 4e is the SEM image of the PbTe sample formed with water/glycerol (3:1 volume ratio) at 140°C for 24 h. It shows clearly the fine particles with similar shape and a size in the range of 70 to 200 nm. The SEM image of the sample prepared with water/glycerol at 200°C for 24 h (Figure 4f) shows larger particles in the range of 200 to 500 nm in various shapes. The SEM results indicate that the particle size increases with the increase in the synthesis temperature when water/glycerol is used as solvent. From the SEM images, it can also be concluded that the combination of water and glycerol gives rise to nanoparticles with similar shape and small size compared to the use of alcohol or water alone as solvents. The use of ethanol or a water/glycerol mixture as solvent yields PbTe nanoparticles with uniform shape and size as compared with the PbTe particles prepared with only water. A report by Zhu et al. [17] also suggests that solvothermal route of synthesis is more favorable than the hydrothermal one due to the strong polarity of the organic material in the solvothermal route which accelerates the dissolution of Te in the reaction process.

**Figure 5:**
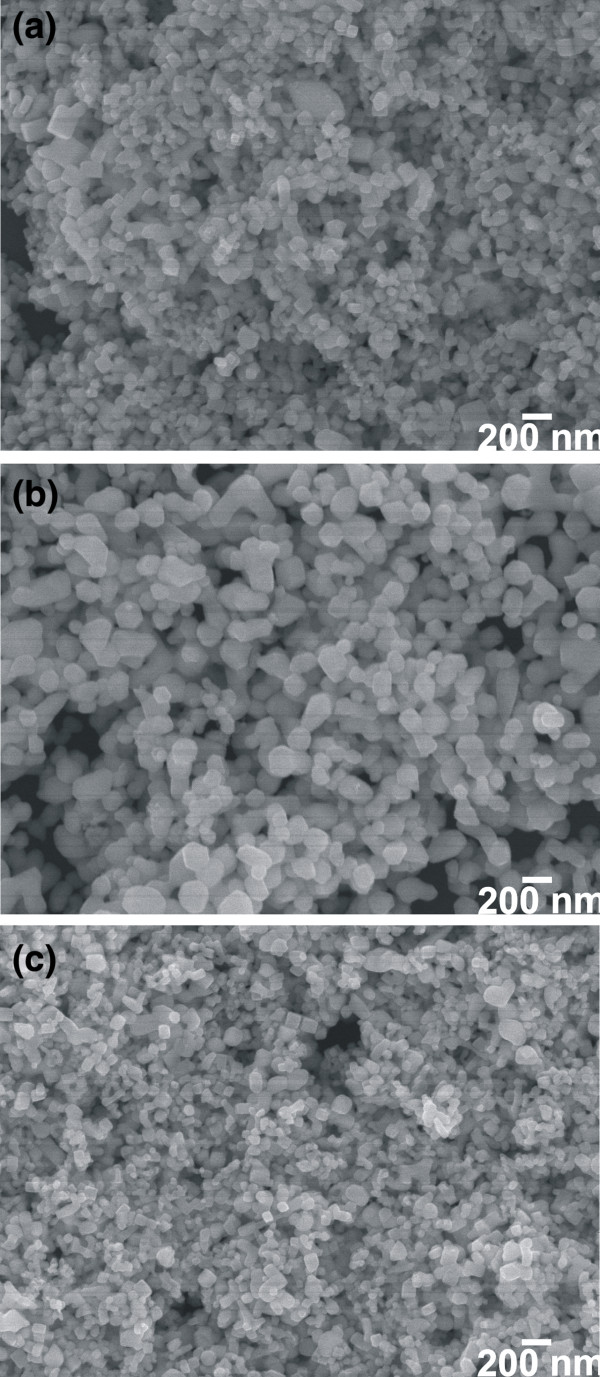
**Effect of use of surfactants on the formation of undoped PbTe.** SEM images of undoped PbTe synthesized with **(a)** CTAB, **(b)** SDS, and **(c)** Triton, respectively, as surfactants in water/glycerol (3:1 volume ratio) solution at 140°C for 24 h.

To further understand the effect of addition of the surfactant on the surface morphology of the PbTe samples, the synthesis process was repeated with a water/glycerol (3:1) solution at 140°C for 24 h using CTAB, SDS, and Triton, respectively, as surfactants. Figure 5a shows the SEM image of the PbTe prepared with CTAB, which indicates the formation of mostly cube-shaped nanoparticles with size in the range of 65 to 145 nm. The sample synthesized with SDS (Figure 5b) shows fewer nanocubes and more irregular nanoparticles compared to the nanoparticles synthesized with CTAB; the size of nanoparticles ranges from 70 to 230 nm. The synthesis of the PbTe sample with Triton (Figure 5c) yields fine particles with the size in the range of 40 to 120 nm. From the SEM images, it can be concluded that the PbTe nanoparticles synthesized at 140°C for 24 h with a water/glycerol solution with the addition of different surfactants (Figure 5) are more uniform in shape and size compared to the nanoparticles synthesized without surfactants (Figure 4e). This can be attributed to the presence of surfactant as a shape-directing agent which is expected to control the size and shape of the particles. PbTe nanoparticles synthesized with CTAB and Triton are smaller in size, while nanoparticles synthesized in SDS are bigger in size which are comparable to the nanoparticles synthesized without surfactants. Zhu et al. [18] reported the synthesis of three-dimensional hierarchical structure of PbTe by a hydrothermal method with or without surfactants using different molar concentrations of NaOH and concluded that the morphology of the PbTe crystals depends on the synthesis temperature, time, and most importantly on the concentration of NaOH. This work also reported the synthesis of PbTe nanoparticles without any hierarchical structure, similar to our PbTe nanostructures, with or without 1 M NaOH at 160°C, and without the use of any surfactants.

**Figure 6:**
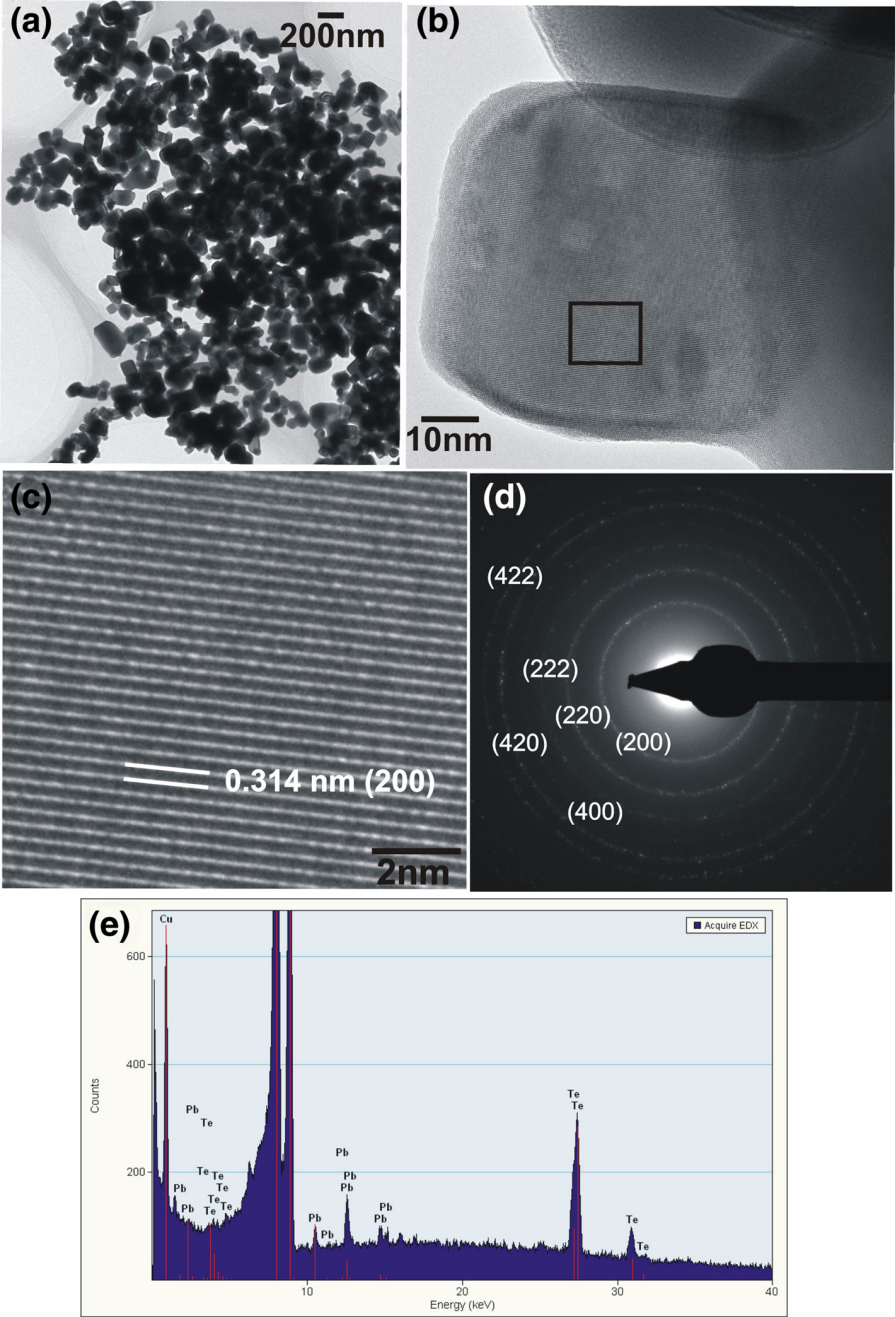
**TEM images of undoped PbTe synthesized without surfactants at 140°C for 24 h with water/glycerol (3:1) solvent. (a)** Low-magnification TEM image, **(b)** high-magnification TEM image, **(c)** HRTEM image of the magnified region indicated by an open box in **(b)**, **(d)** SAED pattern, and **(e)** EDS pattern.

The structure of the as-prepared PbTe sample synthesized at 140°C for 24 h with a water/glycerol solution (i.e., sample PbTe-2, its corresponding SEM image is Figure 4e) was analyzed by TEM, HRTEM, SAED, and EDS. Figure 6a is the low-magnification TEM image of the PbTe nanoparticles with various sizes of 75 to 220 nm. The high-magnification TEM image of the PbTe sample (Figure 6b) indicates that the nanoparticles have cube-like shape. Poudel et al. [26] also reported the cube-like PbTe nano- and microparticles synthesized hydrothermally at 100°C and 160°C, respectively, for 10 h without surfactant. However, with surfactants, various morphologies of PbTe crystals including hierarchical structures were obtained. Recently, PbTe microcubes were prepared using a composite-hydroxide-mediated approach [27]. Instead of using organic or inorganic solvents, this work used a mixture of NaOH and potassium hydroxide (KOH) to yield PbTe microcubes with the size of several micrometers. Figure 6c shows the HRTEM image of the magnified region on the nanocube indicated by the open box in Figure 6b. The HRTEM image indicates equally spaced lattice fringes separated by a distance of 0.314 nm which corresponds to the *d*-spacing of the (200) plane of the cubic PbTe [14]. Figure 6d shows the clearly distinguishable SAED ring patterns which can be indexed to different lattice planes of cubic PbTe. The chemical composition of the PbTe sample was analyzed by an EDS spectrum (Figure 6e) which shows that the as-prepared sample consists of only Pb and Te, hence confirming the chemical purity of the sample. The peak corresponding to Cu in the EDS spectrum arises from the TEM grid used for preparing the TEM specimen. From the TEM analysis, it can be concluded that the clear lattice fringes in the HRTEM image and the distinct rings in the SAED pattern reveal the high crystalline quality of the as-synthesized PbTe nanostructures.

**Figure 7:**
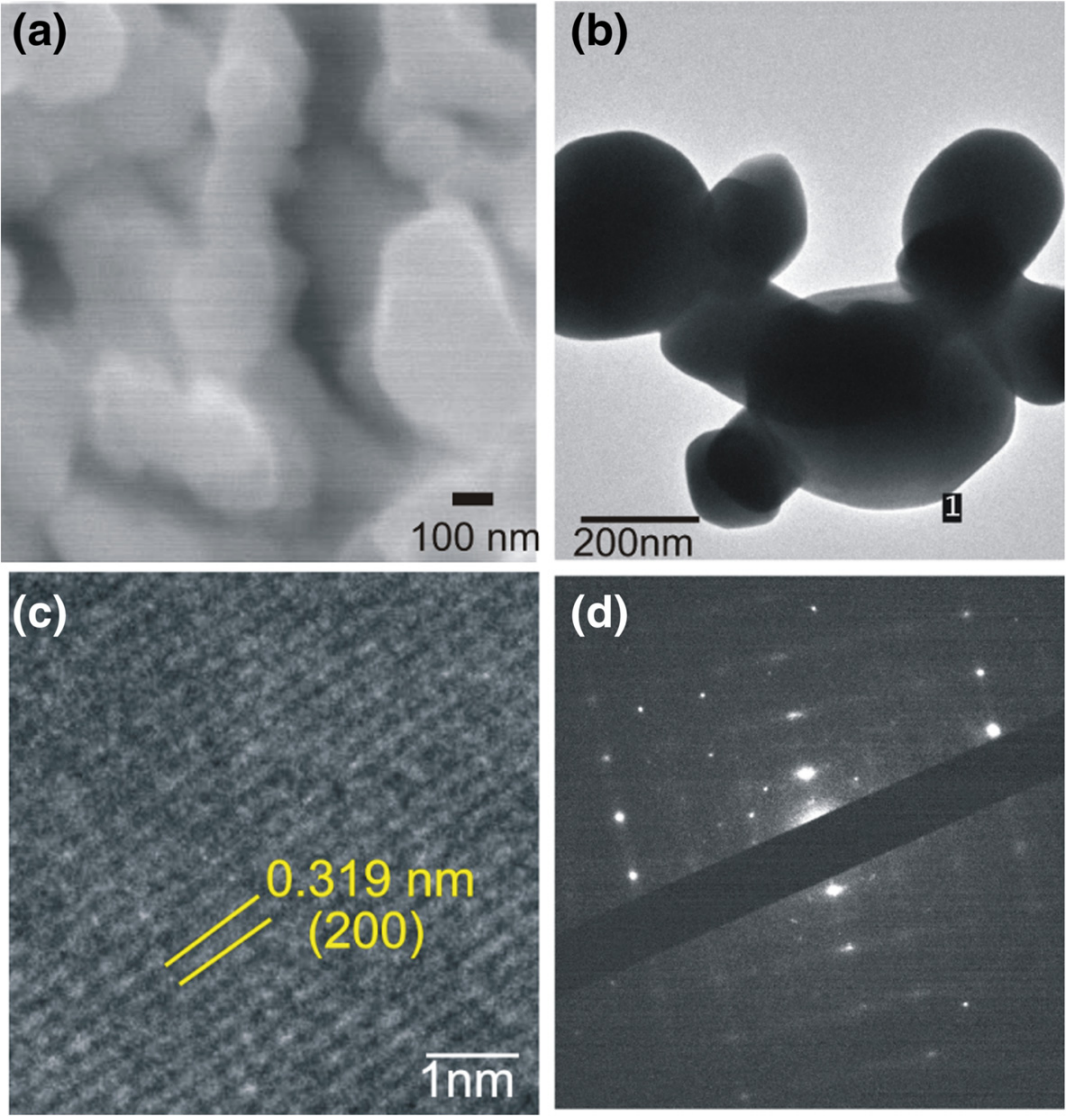
**SEM and TEM images of as-prepared In.**_**01**_**Pb**_**.99**_**Te samples synthesized in water/glycerol solution at 140°C for 24 h (In01PbTe). (a)** SEM image, **(b)** TEM image, **(c)** HRTEM image, and **(d)** SAED pattern.

Surface morphology and structural analyses of the as-prepared In-doped PbTe samples were performed with SEM and TEM examinations, respectively. Since both indium-doped PbTe samples (In01PbTe and In02PbTe) yielded nanoparticles with similar shapes and sizes, only SEM and TEM images of the In01PbTe sample synthesized at 140°C for 24 h in water/glycerol solution is presented in Figure 7. The SEM image (Figure 7a) shows the presence of nanoparticles in various shapes with size in the range of 120 to 250 nm. The nanoparticles are bigger in size as compared to the nanoparticles present in the undoped PbTe sample synthesized at the same conditions (see Figure 4e). The high-magnification TEM image (Figure 7b) of the as-prepared sample reveals the nanoparticles with size of around 150 to 265 nm. Figure 7c shows the magnified region of a nanoparticle as indicated by the letter l in Figure 7b. It shows equally spaced and clear lattice fringes separated by 0.319 nm which is in agreement with *d*-spacing of (200) plane of cubic PbTe. The SAED pattern (Figure 7d) shows the distinguishable diffraction spots which indicate the single-crystalline nature of the In01PbTe cubic structure.

## Conclusion

Undoped and In-doped PbTe nanoparticles were synthesized via the solvothermal and hydrothermal routes with or without surfactant at different preparation conditions. It is found that the solvent plays a very important role in the size and shape of the PbTe and In-doped PbTe nanoparticles. A water/glycerol mixture used as solvent yields nanoparticles with relatively uniform shapes and narrow size distribution, while water used as the solvent will result in nanoparticles with irregular shapes and wide range size distribution. Absence of any impurity phase of indium in the XRD pattern indicated that indium was likely doped into the lattice sites of Pb in PbTe. The presence of multiple indium lines in the LIBS emission spectra for indium-doped PbTe samples, In01PbTe and In02PbTe, confirms the incorporation of indium into the PbTe matrix. The theoretical calculation also indicates that indium is likely to replace lead during the doping process for the smaller concentration of indium (<3 at%) which complements the results obtained from LIBS and XRD analyses. The In-doped and undoped PbTe nanostructures are intended to be utilized in future thermoelectric applications. In-doped PbTe is expected to exhibit enhanced thermoelectric property due to improved electronic properties upon indium doping.

## Competing interests

The authors declare that they have no competing interests.

## Authors' contributions

KK carried out the synthesis process, analyzed the data, and drafted the manuscript. LK participated in the design of the experiment. XW carried out the first principle calculation and revised the manuscript. WL proposed the initial work, supervised the experimental work, and revised the manuscript. PP and JH participated in TEM imaging and image analysis. All authors read and approved the final manuscript.
